# A rare presentation and recurrence of a retroperitoneal Müllerian cyst in a male patient: A case report

**DOI:** 10.1016/j.ijscr.2019.11.001

**Published:** 2019-11-12

**Authors:** Antoine Naem, Ammar Dlewati, Maryam Alhimyar, Mohamad Ali Ousta, Bayan Alsaid

**Affiliations:** a Faculty of Medicine of Damascus University, Syria; b Department of General Surgery of Al-Assad University Hospital, Syria

**Keywords:** RMC, retroperitoneal Müllerian cyst, CT scan, computed tomography scan, CK, cytokeratin, EMA, epithelial membrane antigen, WT1, Wilms tumor 1, PR, progesterone receptor, ER, estrogen receptor, IVC, inferior vena cava, SMA, smooth muscle actin, Case report, Müllerian cyst, Retroperitoneal, Cystadenoma, Paramesonephric cyst

## Abstract

**Introduction:**

The retroperitoneal Müllerian cysts are extremely rare lesions seen mostly in female patients but their occurrence in males is quite possible. Their exact etiology stills unknown, its histological and immunohistochemical characteristics may serve as an evidence that reflects its origin.

**Case presentation:**

We present a case of a resected retroperitoneal Müllerian cyst that reoccurred severely after 3 months of the first laparotomy in a male patient with a significant history of testicular teratocarcinoma. Due to the cyst’s localization and its proximity to vital retroperitoneal structures, total surgical resection couldn’t be made and the recurrence was minimized by post-operative oral chemotherapy.

**Discussion:**

The retroperitoneal Müllerian cyst is a benign cystadenoma that could be confused with other retroperitoneal lesions, which makes the pathological examination with the immunohistochemical study of the cyst’s wall essential to make the diagnosis. The immunopositivity to Cytokeratin 7 and the immunonegativity to Cytokeratin 20 is a key feature that confirm the diagnosis whenever the retroperitoneal Müllerian cyst is suspected. Due to its high vascularity, we highly recommend the administration of chemotherapy which targets the proliferative cyst’s cells.

**Conclusion:**

The RMCs are rare benign lesions that tend to reoccur if total surgical resection isn’t made, when the total resection couldn’t be achieved, the-unspecific anti-mitotic drugs may help in minimizing the recurrence and improve the life quality of the patient.

## Highlights

• The Müllerian cyst incidence in males is rare but quiet possible.
• The pathological and immunohistochemical examination is essential for the diagnosis.
• The immunopositivity for WT1 plays a great role in explaining the cyst’s behavior.
• Applying the total surgical resection is mandatory in preventing the recurrence.
• Chemotherapy can minimize the recurrence and improve the patient’s life quality.

## 1 Introduction

The retroperitoneal Müllerian cysts (RMCs) are rare lesions of the retroperitoneum seen mostly in female patients, one hypothesis suggests that these cysts are embryological remnants of the Müllerian ducts [1], which are a pair of ducts that originate on the lateral aspect of the urogenital ridge during the gestation, in male embryos, they regress under the influence of the Anti-Müllerian hormone secreted by the Sertoli cells of the developing testis, leaving the appendix of the testy and the prostatic utricle as embryological residues. Others indicated that the mesothelium of the peritoneum can undergo a metaplastic transformation into any epithelium of Müllerian origin [2], however, the real etiology of the RMCs isn’t well understood till the moment. In most cases, the diagnosis of the RMCs was confused with other retroperitoneal and abdominal lesions, and the only way to confirm the diagnosis was the pathological examination. Even that RMCs are benign lesions, they tend to grow and reoccur if total surgical resection wasn’t made [3,4], as it is the only curative treatment that has been applied. Here we report a retroperitoneal Müllerian cyst in a 23 years old male with a past medical history of a testicular teratocarcinoma, with a review of the English literature as an attempt to fully describe the clinical, pathological, immunohistochemical characteristics of the RMCs. To our best knowledge, this is the second reported case of a RMC occurring in a male patient [5] and the first reported case of an unresectable RMC due to its sensitive localization. This article has been reported in line with the SCARE criteria [6].

## 2 Presentation of case

**Fig. 1:**
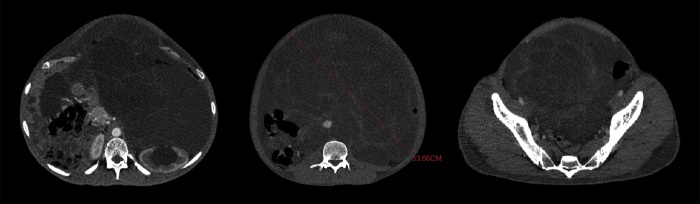
The CT scan that was made before the first operation: showing the huge extension of the cyst occupying all the abdomen, pushing other abdominal organs to the right.

A 23 years old man presented to the emergency department complaining of acute dyspnea, the clinical examination revealed an extremely distended abdomen, a scrotal edema, and grade IV pitting edema in his lower extremities. His past medical history was significant for testicular teratocarcinoma which was treated by orchiectomy followed by a course of eight shots of chemotherapy. A computed tomography scan (CT scan) revealed a large multilocular cystic formation arising from the retroperitoneum, occupying the entire abdomen, displacing the abdominal organs to the right. In addition, a left hydronephrosis was noticed due to compression of the left ureter (Fig. 1).

**Fig. 2:**
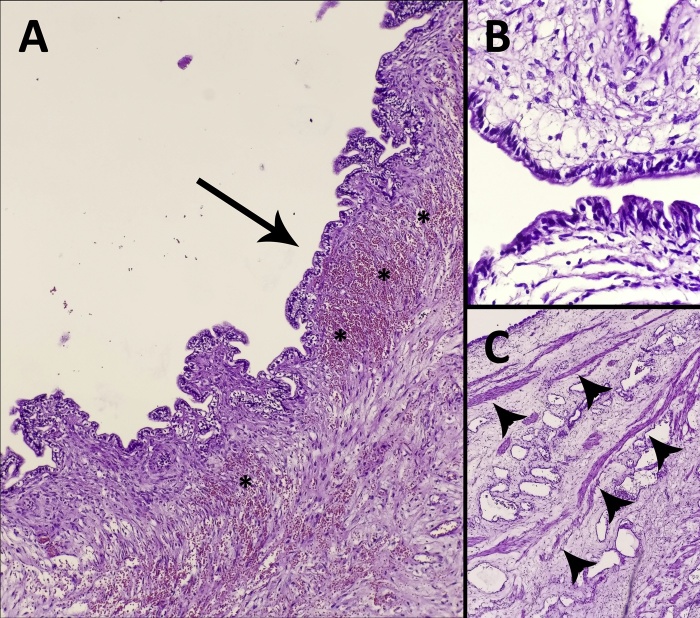
The microscopic appearance of the cyst’s wall (A) showing the ciliated columnar epithelium (arrow), the fibrous stroma beneath it, and the intensive vascularity (*), (B) the single layer of the ciliated columnar epithelium without atypia. (C) showing the smooth muscle bundles within the cyst’s wall (arrow heads).

As the cyst’s nature was not clear and several aspiration attempts failed to relief the symptoms due to the cystic septations, an emergent debulking surgery was performed. A midline incision was made, but due to the large size of the cyst and its close proximity to the retroperitoneal viscera and vasculature, an “en-bloc” resection could not be performed, so aspirating the cystic fluid followed by resecting the cystic septations were the only way to achieve the removal of the cyst from the retroperitoneum. At the end of the operation, 20 liters of serous fluid were aspirated and the majority of the cyst’s septa were resected. Macroscopic examination revealed a multilocular cystic mass composed of smooth surfaces without any papillae or vegetations. Microscopic examination revealed that the cyst was lined with a single layer of columnar epithelium with normal nuclei; no atypia was noticed, beneath the epithelium, a fibrous stroma, smooth muscle bundles, and extensive vasculature were found (Fig. 2).

Immunohistochemically, the cyst’s epithelium was immunoreactive to cytokeratin 7 (CK-7), epithelial membrane antigen (EMA) and Wilms tumor 1 (WT1), and it was immunonegative for the progesterone receptor (PR) and cytokeratin 20 (CK-20), while only the stromal cells were positive for estrogen receptors (ER), therefore, a retroperitoneal Müllerian cyst was diagnosed.

**Fig. 3:**
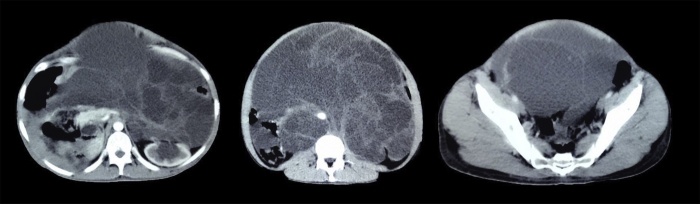
The second CT scan that was made after 3 months of the first operation: showing the aggressive recurrence of the cyst to occupy the abdomen again.

After the surgery the patient was only stable for 3 months before the cyst reoccurred severely to occupy the entire abdomen again. The cyst’s recurrence was confirmed using the CT scan (Fig. 3), in addition to the elevated serum levels of cancer antigen 125 [41.2 IU/ml].

**Fig. 4:**
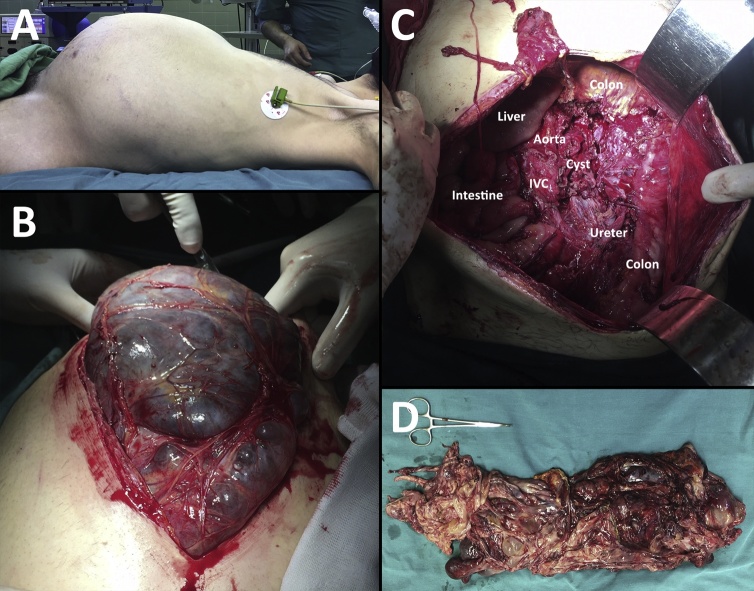
(A) The extremely distended abdomen of the patient before the second operation, (B) Intra-operative view of the cyst showing its huge size and some of its septa, (C) the dissection field after the surgical resection (D): Gross appearance of the müllerian cyst after the resection.

As the benign nature of the cyst was confirmed pathologically, another laparotomy was performed with wider resection field. The inferior vena cava, the abdominal aorta, the kidney and the ureter were heavily isolated and dissected from the cyst’s septa (Fig. 4).

**Fig. 5:**
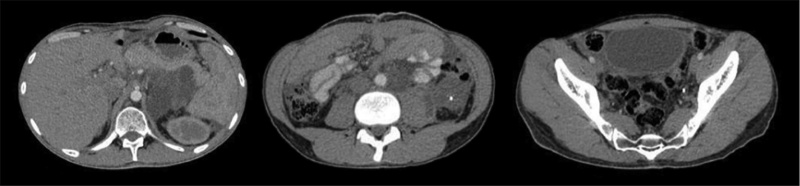
Three slices of the CT scan that was made 6 months after the second surgical resection showing the minimal recurrence of the cyst.

Unfortunately, some parts of the cyst couldn’t be resected in order to protect the main abdominal vessels. The growth, histological and immunohistochemical characteristics of the second resected cyst matched the characteristics of the first specimen. In order to prevent further recurrence, the patient was treated with nonspecific anti-neoplastic drugs (Etoposide 100 mg/day for 5 days and Cyclophosphamide 500 mg/day for 5 days). After 6 months of follow up the patient was stable but a minimal cystic recurrence was detected by the CT scan (Fig. 5).

## 3 Discussion

Retroperitoneal Müllerian cysts (RMCs) are extremely rare lesions of the retroperitoneum which occur mainly in female patients [1,3,4,[7], [8], [9], [10], [11], [12], [13]], but as we have seen in this case, their occurrence in male patients is quiet possible, in most cases, achieving the pre-operative diagnosis is difficult to be accomplished.

In the human embryo, the Müllerian ducts are a pair of tubes that are formed at the lateral aspect of the urogenital ridge during pregnancy. In male embryos, they undergo an incomplete regression under the influence of the Anti-Müllerian hormone secreted by the Sertoli cells of the developing testis, leaving the prostatic utricle and the appendix of testicles as embryological residues, whereas in female embryos, they continue their development to form the major parts of the female reproductive tract.

One hypothesis suggests that incomplete regression of these ducts can leave an asymptomatic remnant which have the capacity of growing under certain hormonal estrogenic stimulations, causing this cystic formation [1], as the majority of patients with RMC didn’t receive any hormonal therapy or suffered from obesity, and our patient is a male with no signs of hyperestrogenemia, we can’t affirm this hypothesis. Another one –which is more accepted- suggests that the peritoneal mesothelium have the ability to differentiate into any epithelium derived from the Müllerian ducts, and the Müllerian cysts are the result of the proliferation of the metaplastic cells, so the peritoneal mesothelium serves as a secondary Müllerian system [2]. In fact, the immunopositivity for WT1 transcription factor may play an important role in confirming the origin of the RMC. In a normal adult, the WT1 is only expressed by the podocytes of the kidney [14], but during the embryogenesis, the WT1 is widely expressed by the mesothelial and submesothelial mesenchymal cells of the coelomic cavity [15] just like the epithelium of the Müllerian cyst, which made us postulate that the RMCs are mimicking the embryological immunohistochemical properties of its origin; the peritoneal mesothelial cells, affirming the hypothesis that mentioned that the re-expression of WT1 may reflect some developmental events [15,16], and subsequently affirming the hypothesis of the secondary Müllerian system [2]. In addition, the abnormal expression of WT1 by tissues that don’t usually express it results in highly proliferative neoplasms, such as the desmoid tumors, i.e. WT1 is considered an oncogene when it’s expressed abnormally [14]. Somehow, this can explain much of the cyst’s behavior and characteristics, but not the full structure of the cyst’s wall.

Whatever the origin of these cysts is, it doesn’t change the fact that the pre-operative diagnosis is quiet hard because the clinical examination lacks of information and the radiologic findings are unspecific.

To the best of our knowledge, the only way to confirm the diagnosis of RMC is examining the pathological and immunohistochemical features of the cyst, some authors considered that the multiple layers of the cyst’s wall in addition to the presence of smooth muscle bundles is a proof that RMCs emerged directly from the Müllerian apparatus [10]. Although the immunohistochemical staining is somehow more specific, it tends to be immunopositive for the CK-7, and immunonegative for the CK-20, in contrast to the colorectal adenocarcinoma which tend to be immunopositive for CK-20 (65.9 %), and immunonegative for CK-7 (82.9 %) [17], ER and PR are usually positive, but their absence doesn’t exclude the diagnosis of RMCs.

Currently, the only curative treatment that has been applied is the total surgical resection, other treatments such as percutaneous aspiration, resulted in the recurrence of the cyst [3,4]. Even though the pathological examination showed benign epithelium, the cyst showed aggressive behavior and recurrence, which allowed us to conclude that the cyst’s cells are highly proliferative and may respond in the same manner as cancer cells to anti-neoplastic agents, however, some patients whom germinal cell tumors relapsed or didn’t respond to other therapies, showed great response to oral etoposide, which make it a favorable treatment of incurable or relapsed neoplasms [18]. We believe that the adjuvant chemotherapy is a good option to prevent the recurrence of the cyst or minimize it but it can’t replace the surgical resection. Finally, the malignant transformation was mentioned only once in the literature [19].

## 4 Conclusion

The RMC is a rare lesion that can occur in male patients. The final diagnosis is made after examining the cyst's wall microscopically and studying its immunohistochemical profile. The cyst's immunopositivity to CK-7 and immunonegativity to CK-20 have an essential role in differentiating it from other retroperitoneal cystic lesions. Some types of the RMCs may have a good vascular supply which encourage the administration of the anti-mitotic drugs to prevent further growth and recurrence.

## Sources of funding

No funding sources.

## Ethical approval

No ethical approval was needed as we didn’t use any experimental drug or new surgical techniques to treat the patient. We combined multiple classical methods of treatment to achieve the best outcome and prevent further recurrence as long as possible.

## Consent

Written patient consent was taken before reporting his case and sharing his pre-surgical, surgical images and the CT scan radiologic images.

## Author contribution

Antoine Naem: wrote the abstract, introduction, discussion, conclusion, provided the language-editing services and all the captions of the figures.

Ammar Dlewati: wrote the presentation of the case, reviewed the literature and designed all the figures.

Maryam Alhimyar: supervised the literature review and data extraction process.

Mohamad Ali Ousta: participated in the surgical treatment and collecting the patient’s data.

Bayan Alsaid: participated in the surgical treatment and supervised the scientific and academic aspects of the manuscript preparation and submission.

## Registration of research studies

N/A.

## Guarantor

A guarantor of data is the corresponding author, Prof. Bayan Alsaid.

## Provenance and peer review

Editorially reviewed, not externally peer-reviewed.

## Declaration of competing interest

No conflict of interests.
